# I Spy in the Developing Fly a Multitude of Ways to Die

**DOI:** 10.3390/jdb6040026

**Published:** 2018-10-22

**Authors:** Alla Yalonetskaya, Albert A. Mondragon, Johnny Elguero, Kimberly McCall

**Affiliations:** 1 Cell and Molecular Biology Program, Department of Biology, 5 Cummington Mall, Boston University, Boston, MA 02215, USA; ayalonet@bu.edu (A.Y.); eelguero@bu.edu (J.E.); 2 Molecular Biology, Cell Biology, and Biochemistry Program, 5 Cummington Mall, Boston University, Boston, MA 02215, USA; albertm@bu.edu

**Keywords:** programmed cell death, apoptosis, non-apoptotic cell death, autophagy, *Drosophila*, ovary, testis, salivary gland, midgut, neuroblast, glia

## Abstract

Cell proliferation and cell death are two opposing, yet complementary fundamental processes in development. Cell proliferation provides new cells, while developmental programmed cell death adjusts cell numbers and refines structures as an organism grows. Apoptosis is the best-characterized form of programmed cell death; however, there are many other non-apoptotic forms of cell death that occur throughout development. *Drosophila* is an excellent model for studying these varied forms of cell death given the array of cellular, molecular, and genetic techniques available. In this review, we discuss select examples of apoptotic and non-apoptotic cell death that occur in different tissues and at different stages of *Drosophila* development. For example, apoptosis occurs throughout the nervous system to achieve an appropriate number of neurons. Elsewhere in the fly, non-apoptotic modes of developmental cell death are employed, such as in the elimination of larval salivary glands and midgut during metamorphosis. These and other examples discussed here demonstrate the versatility of *Drosophila* as a model organism for elucidating the diverse modes of programmed cell death.

## 1. Introduction

### 1.1. Cell Death in Development and Disease

Programmed cell death (PCD) is defined as cell death that occurs normally during development or to maintain cellular homeostasis. PCD falls under the umbrella of regulated cell death, which constitutes all cell death that is controlled through a precise molecular mechanism [1,2]. Examples of PCD in mammalian development include the sculpting of digits [3], the hollowing out of the proamniotic cavity [4], the deletion of structures such as Müllerian duct regression in males [5], and adjusting cell numbers such as neurons where up to 50% of some subtypes die throughout development [6,7].

Dysregulation of cell death can be harmful to an organism; for example too much cell death is often associated with degenerative diseases such as Alzheimer’s disease [8] and amyotrophic lateral sclerosis (ALS) [9]. A failure in cell death can also be detrimental, and is commonly associated with diseases such as cancer [10], autoimmune lymphoproliferative syndrome [11], and rheumatoid arthritis [12]. In the human body, an estimated 200–300 billion cells die each day [13]. Much of this cell death has been assumed to be apoptotic; however, non-apoptotic forms of cell death are found to be physiologically relevant in development and disease [2].

**Figure 1:**
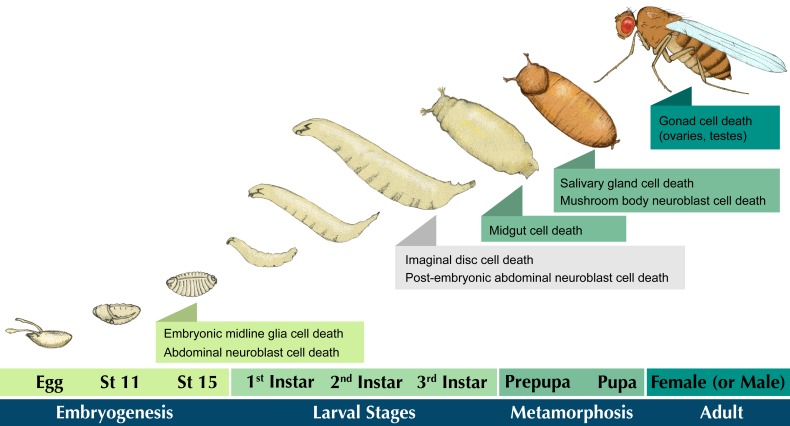
Cell death events during the *Drosophila* life cycle. An illustration of *Drosophila* development. A fertilized egg develops through 17 stages of embryogenesis, culminating in the hatching of a 1st instar larva. The larva molts through two additional stages (2nd and 3rd instar), then undergoes metamorphosis. An adult fly emerges from the pupa and searches for a mate to continue the cycle. Forms of cell death discussed in this review are shaded according to the developmental stage in which they occur. Select examples of other cell death events are listed in gray.

This review focuses on some of the different types of cell death found throughout development in *Drosophila melanogaster*, which may be more relevant to our own physiology than is currently appreciated (Figure 1). We highlight apoptotic death in the embryonic midline glia, abdominal neuroblasts, and mushroom body neuroblast cells. Many other examples of apoptotic cell death, such as in imaginal discs [14], the tarsal region of the leg [15,16], embryonic segment morphogenesis [17], organogenesis of genitalia [17], and cell competition [18], are not reviewed here, and we refer readers to those original publications. We also discuss non-apoptotic modes of cell death with a focus on reproductive organs, and steroid hormone-induced death of larval tissues during metamorphosis.

### 1.2. Types of Cell Death

**Figure 2:**
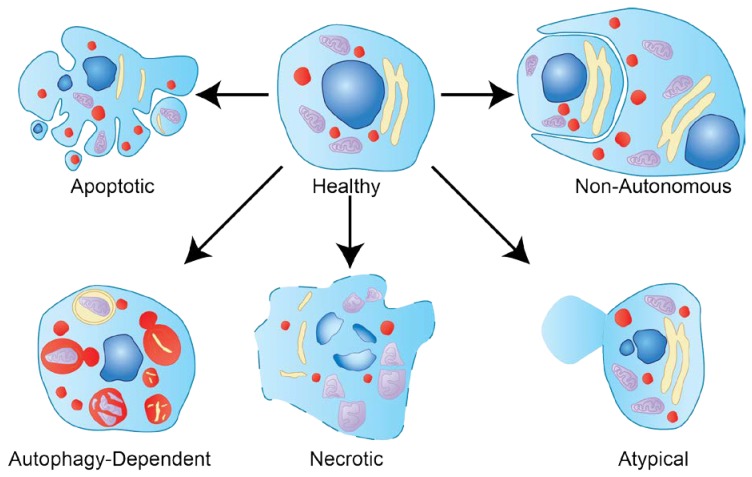
Types of cell death. Diagram of a healthy cell dying by each of the five different classifications of cell death. The apoptotic cell exhibits characteristic blebbing and nuclear fragmentation. Autophagy-dependent cell death is illustrated with numerous acidified compartments and double-membraned vesicles. Necrotic cell death displays plasma membrane lysis and organelle swelling. The atypical form of cell death shown here is pyroptosis; a large pore has formed and plasma membrane contents are spilling out. The non-autonomous cell death demonstrated is phagoptosis, where the phagocyte is utilizing phagocytosis machinery to engulf and eliminate a nearby cell.

Historically, apoptosis has been the most heavily studied form of cell death and has been erroneously used interchangeably with “PCD” [19], since apoptosis is just one form of PCD. There are dozens of other types of cell death: for simplicity, they have been classified into five main categories: apoptotic, autophagy-dependent, necrotic, atypical, and non-cell autonomous cell death (Figure 2) [20]. Each type of cell death is distinguished by the molecular machinery required to initiate and execute it [1].

**Figure 3:**
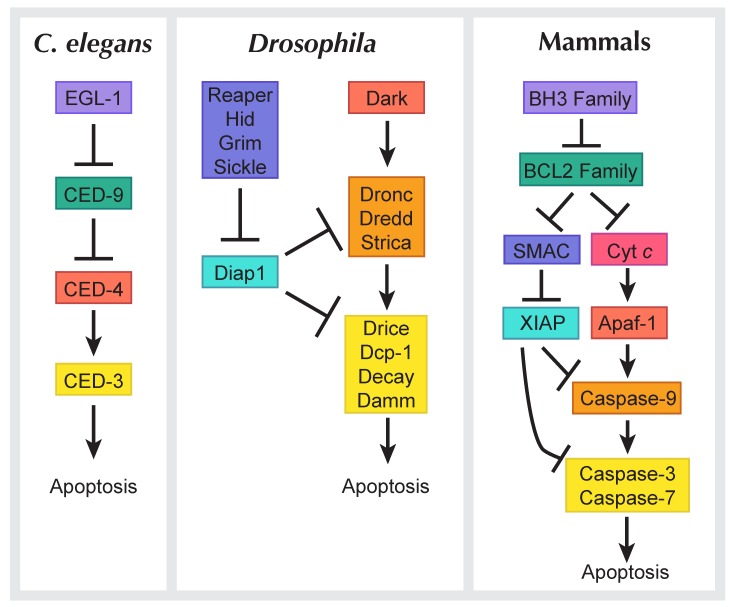
Apoptosis signaling pathways in *Caenorhabditis elegans*, *Drosophila*, and mammals. A simplified schematic comparing models of apoptosis across organisms. Similar families across species are shown in matching colors.

The term apoptosis was first used in 1972 to describe a specific cellular morphology observed in histological samples [21]. About a decade later, the genetic components for apoptosis were identified in *Caenorhabditis elegans*, where the cell lineage of every cell from embryo to adult is known [22,23]. For every adult hermaphrodite, 131 cells are programmed to die throughout development. The discovery of *C. elegans* mutants in which these cells did not die marked the beginning of the genetic characterization of apoptosis [24,25]. These mutants were referred to as cell death abnormal, or “Ced”. Molecular analysis of *C. elegans* and mammalian cell death genes revealed the evolutionary conservation of apoptosis (Figure 3). In *C. elegans*, apoptosis is initiated when Egl-1 (homologous to mammalian BH3-only proteins) binds to Ced-9 (Bcl-2 family) to cause a conformational change, releasing Ced-4 (homologous to mammalian Apaf-1) [26]. Ced-4 subsequently activates the caspase Ced-3, which executes apoptosis [27]. *Drosophila* has a similar molecular program whereby a death stimulus activates the IAP (inhibitor of apoptosis) antagonists Reaper, Hid (Head involution defective), Grim (RHG), and Sickle [28]. IAP antagonists bind to Diap1 (Death-associated inhibitor of apoptosis 1) [29], which unleashes Dronc (homologous to mammalian caspase-9) to associate with Dark (Death-associated APAF1-related killer; Ced-4/Apaf-1), forming the apoptosome [30,31]. The apoptosome activates the effector caspases Drice and Dcp-1 to execute apoptosis [32,33].

In mice, early reports suggested that blocking apoptosis would not produce a viable animal and highlighted many developmental abnormalities [34,35]. Further analysis has revealed that mice can survive without apoptosis genes to adulthood [36] and some of the severe phenotypes were actually a result of other developmental abnormalities not associated with a failure in cell death, with these mice having an appropriate number of neurons and only a partial block of interdigital cell death [37,38]. Such observations warrant a closer examination of the role of non-apoptotic cell death in development. Over the last decade many different types of cell death have been uncovered in a variety of model systems such as *C. elegans*, *Drosophila*, and mice. Some of these newly discovered forms of cell death are highlighted below.

Another form of cell death observed in development is autophagy-dependent cell death. This form of cell death is typically caspase-independent and is associated with the presence of numerous large autophagic vacuoles [2]. In this case, a cell utilizes its own lysosomal machinery to digest itself. The same molecular machinery utilized for macroautophagy is involved in this form of cell death; briefly, autophagosomes encompass large amounts of cytoplasm and/or organelles. Autophagy requires four complexes encoded by distinct groups of autophagy genes: first, the initiation complex composed of the Atg1 complex and its regulators [39]; second, the phosphatidylinositol-3-kinase (PI3K) complex (including Atg6/Beclin 1) responsible for the formation of an isolation membrane or phagophore (also called vesicle nucleation) [40]; third, the Atg8 and Atg12 conjugation system which is required for autophagosome expansion [41]; and fourth, the Atg9, Atg2, and Atg18 proteins which are required for vesicle formation [42,43,44,45,46]. Once formed, the autophagosome fuses with a lysosome and its contents are degraded [47]. It has been suggested that autophagy-dependent cell death is used when large amounts of cell death occur, such as in tissue remodeling [48]. The degradation of the *Drosophila* salivary glands and midgut are well-studied examples of autophagy-dependent cell death [49,50]. In mammals, studies have demonstrated the involvement of autophagy-dependent cell death in the regression of the corpus luteum [51]. It is important to note that autophagy-dependent cell death should not be confused with autophagy that may occur in parallel with cell death [2].

Necrotic cell death is characterized by plasma membrane rupture, organelle swelling, and nuclear condensation [52]. Necrosis had routinely been regarded as a form of accidental cell death, but specific molecular components have been identified for a regulated form of necrosis in mammals called necroptosis (reviewed in [53,54]). Under typical conditions, tumor necrosis factor receptor 1 (TNFR1) recruits TNFR1-associated death domain protein (TRADD) and receptor-interacting serine/threonine protein kinase 1 (RIPK1). Upon further activation, TRADD and RIPK1 complex with FAS-associated death domain protein (FADD) to activate caspase-8 and drive apoptosis. However, in the absence of caspase-8 activity, RIPK1 instead complexes with RIPK3 to form the necrosome [54]. The necrosome recruits mixed-lineage kinase domain-like protein (MLKL), which is phosphorylated by RIPK3. Upon phosphorylation, MLKL oligomerizes and translocates to the plasma membrane to disrupt membrane integrity [55]. An in vivo role for necroptosis has been found in promoting the degeneration of testes in aging male mice. Specifically, active MLKL was found in spermatogonial stem cells of aged male mice and normal degeneration of the testes was shown to be blocked by a chemical inhibitor of necroptosis [56]. None of the canonical core necroptosis-related genes have been identified in *Drosophila*.

Other atypical forms of cell death exist such as ferroptosis, parthanatos, NETosis, lysosome-dependent cell death, and pyroptosis [2]. Each of these forms of cell death utilizes specific molecular machinery and executioner proteins to kill a cell. For example, pyroptosis is morphologically characterized by cellular swelling as a result of large pores forming in the plasma membrane of a cell [57]. In mice, pyroptosis can be activated by the caspase-1 inflammasome, lipopolysaccharide (LPS)-induced activation of caspase-4, -5, and -11, or caspase-3 activity [58,59]. Upon activation, these caspases activate gasdermin D (GSDMD) by cleaving its C-terminal repressor domain [60]. The N-terminal cleavage product of GSDMD, which is sufficient to induce death upon overexpression alone, oligomerizes and localizes to the plasma membrane where it creates pores 10–14 nm in size [60,61]. Pyroptosis occurs in response to bacterial invasion, where cytoplasmic LPS can directly bind and activate caspase-11 leading to GSDMD activation and pyroptosis [62,63,64]. Recent work in mice has identified chemotherapy drugs that are sufficient to induce pyroptosis by activation of caspase-3. In this instance, gasdermin E (GSDME) is activated by caspase-3, causing pyroptosis, although in cells where GSDME is not expressed, apoptosis occurs instead [59]. Blocking GSDME silencing in cancer cells makes them more susceptible to chemotherapy drugs; inversely, the toxicity of chemotherapy drugs to other tissues can be decreased by inhibiting GSDME [59,65]. Altogether, these atypical forms of cell death are currently being molecularly characterized which will allow for further understanding of their physiological contributions.

The final classification of cell death is non-cell autonomous cell death. In this class, a cell promotes the death of another nearby cell. Examples of this type of cell death include cellular cannibalism, phagoptosis, and entosis [20]. In entosis, a cell physically invades a nearby cell, where it will either be destroyed by lysosomal proteases or escape out of the cell [66,67]. Adherens junctions, Rho, and ROCK activity of the invading cell are required for this process. Additionally, the invading cell can replicate or escape the cell, which is unlike a phagocyte engulfing a dead cell [66]. In vitro, glucose starvation may induce entosis of cancer cells by activating AMPK in the invading cell, recycling nutrients from the weakest cells to support the strongest cells [68]. Unlike entosis, phagoptosis requires phagocytic machinery in the phagocyte to engulf and degrade a nearby viable cell [69,70]. Phagoptosis has been well characterized in vitro using neurons and microglia. In co-cultures of neurons and microglia exposed to LPS or amyloid β, neurons respond by exposing “eat me” signals such as phosphatidylserine, initiating their phagoptosis by microglia [71,72]. This induced neuronal phagoptosis can be prevented by inhibiting phosphatidylserine recognition, thereby blocking the bridging protein MFG-E8 or the vitronectin receptor on phagocytes [73]. In *Drosophila*, overexpression of Draper, an engulfment receptor, is sufficient to drive phagoptosis of nearby cells in the ovary [74].

## 2. Cell Death in the Developing *Drosophila* Central Nervous System

*Drosophila* harbor a complex central nervous system (CNS) which, similar to its vertebrate counterparts, relies on PCD to ensure it develops correctly. PCD serves to establish appropriate neuronal and glial cell numbers, neuronal connectivity, and tissue morphology, and, as such, the precise regulation of PCD is essential to generate a functioning CNS. There are many layers of regulation guiding cell death in the nervous system, resulting in a combination of spatial, temporal, and cell identity cues which determine whether a cell lives or dies. Although some instances of cell death occur in a stereotypical pattern, such that the same cell dies in every wild type embryo, other instances maintain a stochastic element by which factors such as functional connections can impact cell death. Furthermore, nearly every cell death event characterized in *Drosophila* CNS development has been reported to be apoptotic; however, whether this is due to a biological phenomenon or an incomplete scope of research remains unclear.

### 2.1. Brief Overview of CNS Development

**Figure 4:**
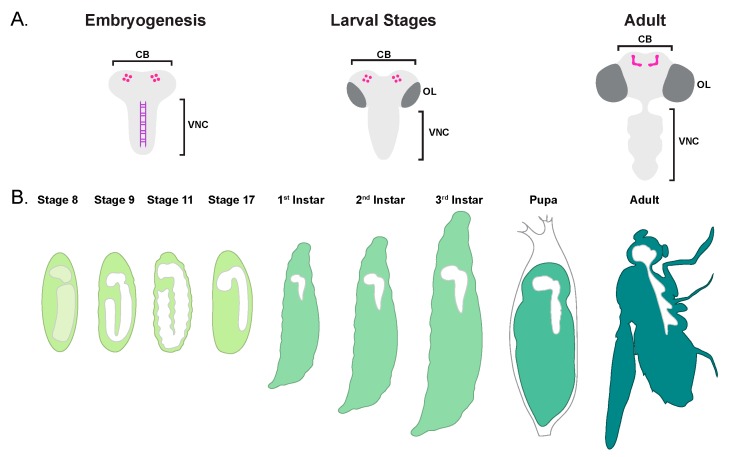
*Drosophila* central nervous system (CNS) development. (**A**) A schematic of the embryonic, larval, and adult *Drosophila* CNS. The central brain (CB), ventral nerve cord (VNC), and optic lobes (OL) are labeled. The embryonic midline is indicated in purple, and the mushroom body neuroblasts, as well as the fully formed mushroom bodies in the adult, are depicted in magenta (not to scale). (**B**) A schematic depicting the sagittal view of the developing CNS. Shown are four embryonic followed by larval, pupal and adult stages. In Stage 8 of embryogenesis, prior to delamination, the neurogenic regions which give rise to the brain and VNC are shown in light green. In subsequent stages, the nervous system is shown in white.

*Drosophila* are holometabolous insects, meaning that they undergo complete metamorphosis (Figure 1). Thus, they require a nervous system capable of functioning in two very different body plans (Figure 4). To do this, the *Drosophila* CNS forms in two waves: one wave of neurogenesis occurs in embryonic stages, and forms the larval CNS [75]. A second wave of neurogenesis takes place in larval and pupal stages, and generates the remainder of the CNS, which will function in the adult. Although some larval neurons persist to adulthood, many are removed by PCD during metamorphosis [76]. Similarly, some neural progenitors are active in both waves of neurogenesis, but many are only active in the first wave, and undergo PCD at the end of embryogenesis [75].

The *Drosophila* CNS can be broken down into three basic components: the central brain, the optic lobes, and the ventral nerve cord (VNC) (Figure 4). The CNS begins to form early in embryogenesis when certain ectodermal cells in the neurogenic regions differentiate into neural progenitor cells, or neuroblasts (NBs) [77]. In Stage 9 of embryogenesis, the central brain and VNC NBs delaminate to the interior of the embryo, where they begin to divide asymmetrically, self-renewing and giving rise to a ganglion mother cell (GMC). Each GMC divides once to become two neurons, two glia, or one of each. These embryonic divisions generate the larval CNS, which is composed of the brain and the VNC. The VNC is segmented into 17 neuromeres featuring a repeating pattern of approximately 30 NBs per hemisegment, each NB giving rise to a specific lineage. Each segment is slightly different, with the most anterior and posterior segments deviating significantly from this pattern [78]. The central brain, on the other hand, has a more complex organization, although some homology to the VNC segments has been proposed [79]. The optic lobes do not form until larval stages, when the neuroepithelial crescents adjacent to the brain give rise to the optic lobe NBs [80].

Embryonic NBs cease to proliferate in the later stages of embryogenesis, either by exiting the cell cycle (especially head and thoracic NBs) or by undergoing PCD (gnathal and abdominal NBs) [81,82,83]. NBs which survive to larval stages are termed “post-embryonic NBs”. They begin to re-enter the cell cycle 8–10 h after hatching, and continue to proliferate to produce the remainder of the adult CNS [76]. Post-embryonic NBs end proliferation at different times and in different ways in a segment-dependent manner [84,85]. Most of the NBs of the central brain and thoracic segments of the VNC end proliferation 20 h into the pupal stages in what is thought to be terminal differentiation [86]. In contrast, post-embryonic abdominal NBs cease proliferation by undergoing PCD in late larval stages [87].

In addition to the PCD of NBs, neurons and glia also die throughout development, both to establish appropriate cell numbers and to remove cells which are no longer required in later stages [88]. There are several waves of neuronal and glial cell death, the major ones being near the end of embryogenesis, late larval through pupal stages, and just after eclosion [89,90,91,92,93]. In the following sections, we examine several examples of PCD which illustrate differing strategies of PCD regulation and execution. For a thorough review of PCD in the developing *Drosophila* CNS, see Pinto-Teixeira et al. [88].

### 2.2. Embryonic Neuroblast Cell Death

At the end of embryogenesis, NBs cease to proliferate by two mechanisms: they either exit the cell cycle and enter a quiescent phase, or they undergo PCD. This PCD was found to be apoptotic, as in the absence of *rpr* and *grim*, embryonic NBs persist and continue to divide, leading to a dramatically enlarged VNC [83]. While most NBs in thoracic segments end proliferation by exiting the cell cycle, most of the abdominal NBs undergo apoptosis [83]. Thus, the regulation of embryonic NB PCD is segment-specific, making it a useful model for understanding the ways in which temporal, spatial, and cell identity cues dictate which cells die and which survive. The mechanism underlying this regulation was primarily elucidated by Arya et al. [94].

Abdominal NB cell identity is established in early embryogenesis by a pulse of the homeotic gene *abdominal A* (*abdA*) [94]. Later in embryogenesis, glial progeny of NBs exposes the Notch ligand Delta on their membranes. Delta binds to and activates Notch on the NB membrane, leading to a second pulse of *abdA* expression. *abdA* triggers cell death by activating Enhancer 1 (Enh1), a cis-regulatory region in the neuroblast regulatory region between *rpr* and *grim*. Enh1 activation drives expression of IAP antagonists *rpr*, *grim*, and *skl*, thus initiating apoptosis.

Mutant and knockdown analyses have untangled the multiple layers of regulation governing embryonic NB apoptosis. Ectopic expression of *Delta* in glia or activated *Notch* in NBs does not induce NB death in thoracic segments, suggesting that abdominal NB cell identity is required for a cell’s competence to induce PCD upon Notch activation. Similarly, ectopic expression of activated *Notch* leads to *abdA* expression only in later stages of embryogenesis, and only in those cells which earlier expressed *abdA*, indicating that both cell identity and temporal cues are required for Notch activation of *abdA*. Additionally, Notch is activated by the expression of *Delta* by the NB’s own progeny; thus, only cells which have appropriately divided are competent to undergo PCD.

Intriguingly, ectopic *abdA* expression is able to activate Enh1, even in thoracic segments; however, this activation does not occur until Stage 13 of embryogenesis, and not in all NBs. How this selective activation is achieved remains to be determined, and is the subject of current research. This may help to elucidate how three abdominal NBs survive the embryonic wave of cell death. Additional open questions in this system include the mechanism by which AbdA activates Enh1, and how the early pulse of *abdA* expression allows for the later induction of *abdA* by Notch activation.

The close regulation of NB PCD allows for a highly stereotyped pattern of apoptosis, giving rise to the intricately patterned VNC in which each segment features a defined set of cells. However, some forms of cell death feature a more stochastic regulation, allowing for the survival of a cell to be determined by its ability to establish functional contacts.

### 2.3. Midline Glia PCD

Midline glia death is an excellent example of stochastic cell death in the embryo. All bilaterally symmetric animals have a boundary along their axis of symmetry known as the midline. In the CNS, this boundary presents a key guidance decision for axons: whether to cross the midline (commissural axons) or remain on one side (longitudinal axons). In the embryonic *Drosophila* CNS, this decision is guided by the neurons and glia that make up the midline. These cells secrete attractive and repulsive cues, which are interpreted by the growing axons [95]. In addition to this guidance role, the midline glia serve to separate and ensheathe the commissural axon tracts, thus ensuring proper wiring of the VNC [96]. Prior to ensheathing axons, a large proportion of midline glia die in a stochastic manner [97].

Midline glia cell death begins in Stage 13 of embryogenesis, prior to which there are approximately ten midline glia per segment. By Stage 17, typically three midline glia remain. Electron microscopy, TUNEL, and the requirement for RHG genes indicate that midline glia die by apoptosis [97]. However, the regulation of this cell death is not uniform. About half of the midline glia require *hid* to die, while the remaining require *reaper* and *grim* [90]. Thus, when *hid* is inhibited, six midline glia persist. This cell death is stochastic; in other words, it is not always the same three cells which survive [97]. The mechanism by which doomed cells vs. survivors are determined provides insight into how a cell’s function can actively shape development.

Axonal contact has long been known to play a role in midline glia survival. Mutants such as *commissureless* and *wrapper*, in which this contact is disrupted, feature excessive midline glia death [98]. In *commissureless* mutants, the few surviving midline glia are found along longitudinal axons, further indicating that axonal contact is protective to midline glia [97]. It was later found that axons promote midline glia survival by secreting the TGFα-like factor Spitz [99]. Spitz binds to EGF receptor (EGFR) on midline glia membranes, activating the Ras/MAPK pathway. MAPK phosphorylates Hid, suppressing its activity and ensuring survival of the midline glia. Thus, only glia which are in direct contact with axons receive sufficient Spitz to inhibit apoptosis.

The contact between glia and axons is established in part by the heterophilic interaction between the cell-adhesion proteins Wrapper (on glial membranes) and Neurexin IV (on neuronal membranes) [100,101]. It has been suggested that there exists a positive feedback loop between *spitz* and *wrapper*, based on the finding that ectopic expression of Spitz leads to activation of a *wrapper* enhancer. This may lead to stronger adhesion between the glia and axon, leading to more Spitz signal reaching the glia, promoting survival [102].

This model for axonal contact-dependent glial survival was predominantly determined by studies in which living midline glia were counted at the end of embryogenesis; however, newer studies using time lapse imaging have uncovered a more dynamic process. Occasionally, a midline glia already in close proximity to axons will die and allow another to take its place [102]. It is not clear whether this death is also due to insufficient Spitz signal, or if another layer of regulation exists.

### 2.4. Mushroom Body Neuroblast PCD

The *Drosophila* mushroom bodies are a pair of structures in the brain which function in olfactory learning and memory. Each mushroom body consists of approximately 2000 intrinsic Kenyon cells [103], as well as non-intrinsic interneurons which project to other brain regions [104]. The entire mushroom body neuronal population is generated by a total of eight NBs (four per hemisphere) [81]. These mushroom body NBs provide a useful example of cell death which may be executed by multiple cell death modalities, although more research is needed to confirm this.

MB NBs are the longest proliferating neural progenitors in *Drosophila*. They divide from embryonic stages until 72 h after puparium formation (APF), when they undergo a dramatic reduction in cell size, followed by a decrease in mitotic activity [105]. At 96 h APF, no mushroom body NBs can be detected, indicating that they have either died or differentiated. It was previously thought that all central brain NBs end proliferation through terminal differentiation; however, more recent findings indicate that mushroom body NBs die apoptotically around 90 h APF [105]. Morphological evidence for the apoptotic nature of this death includes features such as the presence of activated caspases and fragmented DNA, and the absence of Lamin. Furthermore, upon knockdown of RHG genes or overexpression of the baculovirus pan-caspase inhibitor *p35*, mushroom body NBs can be seen 3–5 days after eclosion of the adult. The role of RHG genes was further examined using flies *trans*-heterozygous for the *Df(3L)H99* and *Xr38* deletions, which renders them homozygous null for *rpr* and heterozygous for *hid*, *grim*, and *skl*. In these mutants, mushroom body NBs persist in the brain up to a week after eclosion. This persistence is not indefinite, however, as no mushroom body NBs are seen in RHG-deficient flies two weeks after eclosion. Interestingly, the persistence of mushroom body NBs is extended to one month in flies in which the Insulin/PI3K or autophagy pathways are inhibited in addition to RHG genes. This suggests that autophagic cell death may be able to remove aberrant mushroom body NBs resulting from a failure in apoptosis. Autophagy and insulin signaling also appear to play a role in the death of mushroom body NBs even in the presence of apoptosis, as *foxo* mutants, as well as flies expressing dominant negative Atg1 in mushroom body NBs, feature a delay in mushroom body NB removal. More research is needed to determine whether autophagy is important for the timing of mushroom body removal, or if it is contributing to the cell death process.

## 3. Death in Reproductive Systems

### 3.1. Developmental Phagoptosis in the Drosophila Ovary

**Figure 5:**
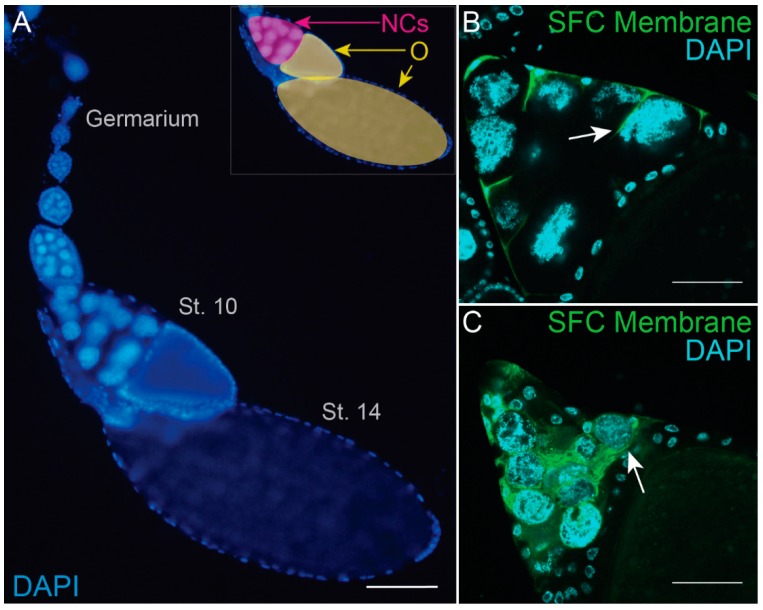
The *Drosophila* ovary. (**A**) An image of a single ovariole stained with DAPI to label DNA. Oogenesis begins in the germarium and ends with a fully developed Stage 14 egg chamber. During the late stages of oogenesis, nurse cells (NCs) dump their cytoplasm into the oocyte and are eliminated. This dramatic change is readily seen when comparing the Stage 10 egg chamber in which NCs take up about half of the egg chamber (inset, pink region) to Stage 14 when NCs have been eliminated (inset, yellow region). (**B**) Stretch follicle cell (*SFC*) > *Myr-GFP* Stage 11 egg chamber stained with DAPI. SFC membranes (green, arrow) begin to surround NCs as they dump their cytoplasmic contents into the oocyte. (**C**) *SFC* > *Myr-GFP* Stage 13 egg chamber stained with DAPI. SFC membranes (green, arrow) completely surround NCs. All scale bars = 50 µm.

The *Drosophila* ovary is composed of ~15 ovarioles containing progressively developing egg chambers (Figure 5A). Each egg chamber consists of 15 nurse cells connected to the oocyte through intercellular bridges as a result of incomplete cytokinesis. The germline cyst is surrounded by an epithelial follicle cell layer. Every egg chamber matures through 14 morphologically distinct stages of oogenesis, producing a fully developed egg at Stage 14 that can be fertilized and oviposited. Throughout development, the nurse cells generate proteins, organelles, and RNA to transport into the oocyte before undergoing cell death in the final stages of oogenesis. Importantly, the *Drosophila* ovary is a closed system without macrophages to clear corpses [106,107].

Of particular interest, the nurse cells undergo several specialized events before dying and being cleared in late oogenesis. This developmentally regulated cell death is highly reproducible and occurs in every egg chamber—for every fully developed egg, all 15 nurse cells die. The first sign of nurse cell death is nuclear membrane permeabilization which occurs at the end of Stage 10 [108]. At Stage 10B-11, a cytoplasmic actin network surrounds each individual nurse cell nucleus as each cell transfers its cytoplasmic contents into the oocyte through a mechanism termed “dumping” which is complete by Stage 12 (reviewed in [109,110]).

Following dumping, the nurse cell nuclei condense and the remnants are cleared between stages 12-14. Early reports suggested that nurse cells undergo apoptosis [111,112]; however, further examination has demonstrated that apoptosis does not play a major role in nurse cell death. Specifically, nurse cells die normally even if they are lacking the canonical apoptosis inducers (*reaper*, *hid*, and *grim*), caspase initiators (*dronc* or *strica*), or if apoptosis inhibitors (p35 or Diap1) are overexpressed [113,114,115]. Moreover, even the combined inhibition of apoptosis and autophagy does not prevent nurse cell death [116]. These findings indicate that nurse cells die by another cell death mechanism.

The stretch follicle cells, a layer of squamous epithelial cells, are a specialized subset of ~50 follicle cells that cover the 15 nurse cells on the anterior side of the egg chamber (reviewed in [117]). Between Stages 11 and 12, the stretch follicle cells completely surround and encompass the individual nurse cells [118] (Figure 5B,C). In Timmons et al. [118], genetic ablation of stretch follicle cells was shown to block nurse cell dumping, death, and clearance, highlighting the significant role of stretch follicle cells in orchestrating nurse cell death events.

Further investigation into the failure of dumping in egg chambers where stretch follicle cells have been genetically ablated revealed a lack of nurse cell cytoplasmic actin bundles which normally holds the nurse cell nuclei in place. Without the cytoplasmic actin, the nurse cell nuclei become stuck in the ring canals, blocking dumping from progressing [118]. The signaling molecules or mechanism through which stretch follicle cells induce nurse cell cytoplasmic actin has not yet been elucidated. Nurse cell dumping and death are two separate events; nurse cells in egg chambers mutant for genes that inhibit dumping will continue to die, although the death is delayed [113,119].

Later events of nurse cell death are associated with the phagocytosis machinery. Draper, an engulfment receptor, becomes enriched on stretch follicle cell membranes at Stage 11 and strongly accumulates by Stage 12 [118]. In Stage 12, acidic vesicles, labeled by LysoTracker, can be found in the stretch follicle cells [118]. Additionally, JNK signaling is activated in the stretch follicle cells during Stage 12. By Stage 13 the nurse cells become completely acidified, and DNA is fragmented. By Stage 14 all nurse cells are completely cleared [118].

Disruption of engulfment genes (*draper*, *Ced-12*, *Eato*, *α-PS3*, *βPS*, or the JNK pathway) in stretch follicle cells blocks multiple cell death events including nurse cell acidification, DNA fragmentation and clearance [118,120,121]. Blocking the ABCA transporter Eato (Engulfment ABC transporter in the ovary) in stretch follicle cells additionally prevents some of the follicle cells from completely surrounding the nurse cells [121]. However, knockdown of *draper* or *ced-12* does not block stretch follicle cells from completely surrounding the nurse cells, but does block nurse cell acidification [118].

Lysosomal trafficking genes *spinster* and *deep orange* are required for nurse cell elimination, suggesting a role for lysosome genes in the acidification and degradation of nurse cells [118,122,123]. Recent findings have revealed that stretch follicle cells surround the nurse cells and subsequently recruit V-ATPases and chloride channels to the plasma membrane to extracellularly acidify the nearby nurse cells. Following nurse cell acidification, the stretch follicle cells release cathepsins to eliminate the nurse cells [124].

Altogether, it is likely that the direct contact of stretch follicle cells with the nurse cells and the lysosomal machinery of the stretch follicle cells drive nurse cell death through phagoptosis. Nurse cell phagoptosis serves as an efficient and precise mechanism to both kill and clear a group of cells after their developmental duties are complete without harming the nearby oocyte.

### 3.2. Non-Apoptotic Cell Death in the Drosophila Testis

The *Drosophila* testis offers an excellent opportunity to study another non-apoptotic form of cell death. The *Drosophila* male reproductive system is composed of two testes, each in the shape of a coiled tube. The apical tip of the tube houses the stem cell niche which produces spermatogonial cysts, each composed of 16 spermatogonial cells (or spermatocytes) surrounded by two cyst cells [125]. Twenty to thirty percent of spermatogonial cysts undergo PCD [126]. The surviving spermatogonial cysts continue to develop through spermatogenesis by undergoing meiosis and subsequently differentiating to create 64 elongated spermatids. The spermatids undergo nuclear condensation and expel most of their cytoplasm into a “waste bag” [125]. Interestingly, caspases have a non-apoptotic role in sperm development, where caspase activity is required for the removal of cytoplasm [127]. The spermatids continue to mature and are released from the basal end of the testis into the seminal vesicle [125].

The 20–30% of spermatogonial cysts that undergo PCD do not have detectable caspase activity, and inhibition of caspases by overexpression of *p35* or *Diap1* paradoxically leads to an increase in spermatogonial cyst cell death [126]. Yacobi-Sharon et al. [126] performed a genetic screen and found the mitochondrial serine protease high-temperature requirement A2 (Htra2/Omi) to be necessary for spermatogonial cyst cell death. Further analysis demonstrated that the protease activity, and not the signaling activity of Htra2/Omi is required, although it is unclear what direct role Htra2/Omi is performing in these dying spermatogonial cysts [126]. *Drosophila* Bcl-2-like proteins, Debcl and Buffy, are also required, likely for the release of Htra2/Omi from the mitochondria [126].

Morphological examination of spermatogonial cyst cell death by electron microscopy shows that cells exhibit apoptotic hallmarks such as cellular shrinkage and chromatin condensation, but nuclei are also increasingly crenellated and maintain their nuclear membrane until late stages of degradation [126]. A recent report has raised the possibility that spermatogonial cyst cell death may be necrotic, based on morphological examination and propidium iodide staining [128].

Disruption of autophagy by blocking *Atg8*, *Atg7*, or *Atg1* does not have any effect on spermatogonial cyst cell death [126]. However, disruption of lysosomal trafficking genes *deep orange* (*dor*) and *carnation* (*car*), as well as cathepsin D (*cathD*) and acid DNase II (*DNaseII*), leads to a decrease in spermatogonial cyst cell death. Additionally, LysoTracker staining revealed that the entire dying cyst becomes acidic, suggesting an important role for lysosomes in the execution of spermatogonial cyst cell death [126]. Interestingly, developmental PCD in both the ovary and testis require lysosomal trafficking genes, exhibit an increase of LysoTracker staining, and do not require autophagy genes. Further work characterizing the molecular pathway and potential role of lysosomes should be done to better understand this alternative cell death pathway.

## 4. Steroid Hormone-Induced Cell Death in the Elimination of Larval Tissues

### 4.1. Removal of the Larval Salivary Glands

Often, a cell undergoing PCD seems to be dominated by a single cell death program. However, several cases have been identified in which molecular components from different cell death modalities collaborate to eliminate cells. *Drosophila* provides at least two robust systems in which to study this phenomenon. During oogenesis, environmental insults, such as nutrient starvation or predator exposure, increase the rate of cell death in mid-stage egg chambers [129,130]. The degradation of the germline cyst relies on both apoptotic and autophagic genes, and is further described in Jenkins et al. [131]. Another instance occurs in *Drosophila* development and involves the degradation of larval salivary glands in metamorphosis. This process happens in response to steroid hormone signaling, and also depends on apoptosis and autophagy genes [132].

Salivary glands form early and relatively quickly in embryogenesis, from about 4.5–10 h of development [133]. The tissue is composed of just two cell types, duct and gland, that differentiate from a primordium of about 100 cells in parasegment 2 [133,134]. These cells migrate within the embryo to establish two salivary gland tubes with individual ducts that merge at a common duct connected to the larval mouth [134]. At the end of the third instar, larvae stop eating and begin wandering in search of a suitable site to pupate. The lumen of the salivary glands bloats with a liquid secretion. As larvae slow and begin to pupariate, this glue secretion is expressed through the ducts and out the mouth, adhering puparia in place [135].

**Figure 6:**
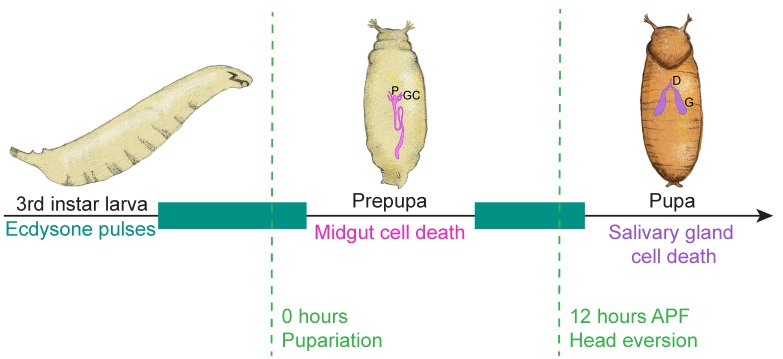
Ecdysone-induced cell death in larval tissues during *Drosophila* metamorphosis. A schematic depicting pulses of ecdysone (teal) during the developmental progression from larva to pupa. The first pulse of ecdysone in late third instar larvae triggers midgut histolysis (pink), including the proventriculus (P) and gastric caeca (GC). The second pulse of ecdysone, ~10 h after puparium formation (APF), leads to salivary gland degradation (purple), including the duct (D) and gland (G) tissues. Figure adapted from Baehrecke [132]. The midgut at 0 h in Jiang et al. was used as a reference for the anterior midgut region in this schematic [145]. Organ location and size approximated.

These and other developmental changes in *Drosophila* are orchestrated by the steroid hormone ecdysone [136,137]. Ecdysone is primarily produced in the prothoracic gland and is converted to the active form, 20-hydroxyecdysone, by P450 monooxygenase in the hemolymph (both forms are often referred to as simply ecdysone) [137]. The receptor for ecdysone is a heterodimer of the nuclear hormone receptors Ecdysone receptor (EcR) and Ultraspiracle (USP) [138,139,140,141]. A pulse of ecdysone in third instar larvae stimulates the transition to prepupal development (Figure 6), and promotes the transcription of early puff genes including *Broad-Complex* (*BR-C*), *E74*, and *E75* in salivary glands [132]. During the prepupal stage, certain larval tissues are destroyed, such as the midgut and anterior muscles, while others persist [132]. As the ecdysone titer diminishes in mid-prepupae, the nuclear hormone receptor *βFTZ-F1* is induced in salivary glands. βFTZ-F1 serves as a competence factor and guides the response in salivary glands of the second ecdysone pulse, which occurs 10–12 h APF, promoting the expression of *BR-C* and *E74* early genes, and *E93* [142,143,144]. This second pulse of ecdysone prompts destruction of further larval tissues, including the salivary glands.

Salivary gland degradation is a powerful model for elucidating the dynamics of multiple cell death programs working within the same cell. Salivary glands degrade rapidly and remnants are difficult to detect 16 h APF [50,145]. Features of both apoptosis and autophagy were observed in early studies including nuclear permeability, DNA fragmentation, caspase activity, and cytoplasmic vacuolization [145,146,147,148]. Notably, blocking both apoptosis and autophagy results in a more severe phenotype than blocking components of either pathway alone [50].

Apoptotic genes are upregulated just prior to the demise of the salivary glands: *rpr* and *hid* are induced 12 h APF, coinciding with the second pulse of ecdysone [145]. The ecdysone receptor complex can directly regulate *rpr* expression by binding to at least one site in its promoter region [149]. Additionally, steroid regulated genes can also affect levels of apoptotic proteins. *BR-C* is required for *rpr* and *hid* expression, while E74A is required for the maximal *hid* expression [149]. E93 is also required for appropriate expression levels of *rpr*, *hid*, *crq*, *Dark*, and *Dronc* [150]. Blocking caspase activity by expressing baculovirus *p35* impairs salivary gland degradation, resulting in persisting tissue even 24 h APF [145,146,148]. However, this persisting salivary gland tissue showed evidence of partial degradation, indicating that caspases were not solely responsible for salivary gland elimination.

Autophagy-dependent cell death is defined by the requirement for autophagy genes or components in promoting cell death events, not merely as bystanders associated with a dying cell [2]. Several autophagy genes are upregulated during salivary gland histolysis [151,152,153]. Importantly, blocked autophagy in *Atg8a* mutant flies does not affect the development of larval salivary glands, but impairs their degradation [50]. Salivary gland fragments persisted 24 h APF and appeared vacuolated [50]. Heat shock-induced pupal expression of *GFP-dAtg8a* in *Atg8a* mutants was sufficient to rescue this phenotype [50]. Additional experiments using autophagy gene mutants or knockdowns further support the requirement of autophagy genes in salivary gland removal [50,154]. Interestingly, the engulfment receptor Draper was found to act upstream of autophagy in dying salivary glands [155]. Draper is cell-autonomously required for salivary gland degradation, but not for the maintenance of the fat body during starvation-induced autophagy, thereby potentially distinguishing the roles of autophagy during cell survival versus death [155]. Furthermore, macroglobulin complement-related (Mcr) was recently shown to act non-cell autonomously and upstream of Draper to regulate autophagy in dying salivary glands—an exciting finding implicating an immune signaling component in regulating autophagy during cell death [156].

Salivary glands are large, polytene cells [157]. Thus, utilizing both apoptosis and autophagy-dependent cell death programs may aid in efficiently breaking down these large cells. It would be interesting to determine the extent to which other polyploid cells also require multiple cell death programs (e.g., mid-stage death of developing egg chambers), or die by non-apoptotic means (e.g., larval midgut). PCD modalities are largely conserved across organisms, so it would be further interesting to determine if there is conservation of cell death modalities in polyploid cells in mammals (e.g., hepatocytes). Moreover, apoptotic cells are typically cleared in vivo by phagocytes [13]. However, no phagocytes have been identified for degrading larval salivary glands [146,156]. Therefore, autophagy-dependent cell death may provide a mechanism for clearing degraded debris at least partially in this system.

### 4.2. Removal of the Larval Midgut

The initial pulse of ecdysone in late third instar larvae heralds the next developmental stage in the *Drosophila* life cycle: metamorphosis. As such, it is a harbinger of death to larval structures. A subset of larval organs undergo histolysis as a result of this first pulse of ecdysone, including the midgut [145,158]. Changes in midgut morphology occur quickly APF, although the timing varies depending on the experimental technique [49,145,159]. In paraffin embedded histology sections, the adult gut epithelium is seen around the larval midgut as early as 2 h APF [159]. At 4 h APF, the proventriculus and gastric caeca, anteriorly adjoining the midgut, contract, and by 6 h APF they are no longer detected with this technique [159]. At 12 h APF, the degenerating larval midgut appears condensed, enclosed within the lumen of a distinct adult midgut [159]. The degraded larval midgut forms the yellow body, which is excreted as the meconium soon after the adult fly ecloses [145].

At first glance, the cell death of the larval midgut seems to resemble that of the salivary glands: the death of both tissues is preceded by growth arrest [50,160], and features of apoptosis and autophagy are also observed in the degenerating midgut [145,148]. As in salivary glands, *rpr* and *hid* expression is induced in the midgut at puparium formation, just before midgut cell death [145]. Additionally, blocked autophagy in *Atg2* and *Atg18* mutants, or knockdown of *Atg1* and *Atg18*, delays midgut cell death [49]. Early experiments reported that blocking caspase activity also blocked midgut degradation [132,145], similar to salivary glands. However, it has since been shown that although caspase activity is detected, caspases are not required for the developmental death of the larval midgut [49]. In further contrast to salivary gland degradation, combined inhibition of autophagy and caspases does not exacerbate the delay in midgut degradation [49]. Thus, while autophagy genes are critical to cell death in salivary glands and the midgut, caspases are essential for histolysis of the salivary gland, but not the midgut [49,50,161].

The midgut provides a number of intriguing avenues for research. Several reports have indicated diverging requirements of autophagy components in driving autophagy-dependent cell death in the midgut versus starvation-induced autophagy in the fat body [44,162]. It remains to be determined how autophagy is regulated in these different biological contexts. Recent work sheds some light on this topic, identifying a novel role for the morphogen Decapentaplegic (Dpp) in larval midgut death through regulating autophagy [163]. Additionally, blocking autophagy only delays developmental midgut death, but compensatory or alternative degradation mechanisms have yet to be revealed. Finally, in salivary glands, caspases have lethal and non-lethal roles determined by spatially restricted interactions with adaptor proteins *tango7* and *Dark*, and varying degrees of *rpr* induction [164]. While caspases are not critical for midgut death, they are active and it would be of interest to ascertain the non-lethal functions of their activity.

Surprisingly, not all larval tissues depend on non-apoptotic modes of cell death. Autophagy genes do not play a role in the death of abdominal muscle cells, which die apoptotically [165]. In general, larval tissues provide an array of models for investigating steroid-induced cell death.

## 5. Conclusions

While cell death was historically thought of as simply a final punctuation to the vibrant life of a cell, it is now appreciated that the process of cell death is equally dynamic. Over a dozen cell death modalities have been identified since apoptosis was discovered to be genetically encoded in *C. elegans* ~35 years ago. The field continues to expand as novel instances and potentially novel forms of cell death are reported. Going forward, it is critical to integrate analyses of morphological, genetic, and biochemical features of dying cells to avoid mischaracterization. Historically, the presence or activity of some apoptotic core machinery had been used to categorize cell death events as apoptotic. However, caspases have a number of non-apoptotic roles in the cell, including cell migration and remodeling [164,166,167], which can confound experimental interpretation. Additionally, as mentioned in the Introduction, caspase activity can also be involved in driving non-apoptotic cell death, such as pyroptosis.

Model organisms have been essential for expanding our understanding of PCD. Although apoptosis was first classified according to morphology observed in mammalian tissue samples [21], model organisms allowed us to unravel the genetic basis of apoptosis [24]. Model organisms continue to propel cell death research by offering key insights. One example is the non-apoptotic cell death of the linker cell in *C. elegans* [168]. In addition to not requiring apoptotic machinery, the degeneration of linker cells is morphologically distinct from apoptosis; rather than nuclear fragmentation, the nucleus crenellates (indents) [168]. Interestingly, similar nuclear changes are apparent in many other examples of developmental cell death including the *Drosophila* ovary [109,169]. Further investigation into the extent of conservation of this morphological event, as well as its potential contributions in the breakdown of the nucleus during cell death is warranted. More broadly, the coordinated analysis of the morphology and molecular biology of cell death in vivo will provide further insight into the diversity of cell death modalities in development.

Outstanding questions:

1. Why are certain PCD modalities favored in certain tissues? For example, cell death in the nervous system seems to occur through apoptosis, while non-apoptotic forms of cell death are observed in reproductive systems.
2. Is there crosstalk between cell death programs, and how does that conversation occur? For example, when one form of cell death is blocked, the cell may still die through an alternative (or secondary) cell death mechanism. In addition, in some instances two different cell death programs contribute to the elimination of cells or tissue, as in larval salivary glands.
3. To what extent are non-apoptotic modes of cell death conserved across species? For example, which types of non-apoptotic death are (or are not) conserved among organisms, and how similar are the signaling pathways?
